# Mortality and Pulmonary Embolism in Acute Respiratory Distress Syndrome From COVID-19 vs. Non-COVID-19

**DOI:** 10.3389/fmed.2022.800241

**Published:** 2022-03-04

**Authors:** Demetrios J. Kutsogiannis, Abdulrahman Alharthy, Abdullah Balhamar, Fahad Faqihi, John Papanikolaou, Saleh A. Alqahtani, Ziad A. Memish, Peter G. Brindley, Laurent Brochard, Dimitrios Karakitsos

**Affiliations:** ^1^Department of Critical Care Medicine, Faculty of Medicine and Dentistry, The University of Alberta, Edmonton, AB, Canada; ^2^Critical Care Department, King Saud Medical City, Riyadh, Saudi Arabia; ^3^Department of Medicine, The Johns Hopkins University Hospital, Baltimore, MD, United States; ^4^Research and Innovation Center, King Saud Medical City, Riyadh, Saudi Arabia; ^5^Department of Critical Care, Keenan Research Center and Li Ka Shing Institute, St. Michael's Hospital, Toronto, ON, Canada; ^6^Interdepartmental Division of Critical Care Medicine, Institute of Medical Science, University of Toronto, Toronto, ON, Canada; ^7^Institute of Medical Science, University of Toronto, Toronto, ON, Canada; ^8^Department of Internal Medicine, University of South Carolina, School of Medicine, Columbia, SC, United States; ^9^Critical Care Department, Keck School of Medicine, University of Southern California, Los Angeles, CA, United States

**Keywords:** acute respiratory distress syndrome, pulmonary embolism, recruitment inflation ratio, ventilatory ratio, COVID-19, respiratory mechanics, interleukin-6 (IL-6)

## Abstract

**Purpose:**

There may be a difference in respiratory mechanics, inflammatory markers, and pulmonary emboli in COVID-19 associated ARDS vs. ARDS from other etiologies. Our purpose was to determine differences in respiratory mechanics, inflammatory markers, and incidence of pulmonary embolism in patients with and without COVID-19 associated ARDS admitted in the same period and treated with a similar ventilation strategy.

**Methods:**

A cohort study of COVID-19 associated ARDS and non COVID-19 patients in a Saudi Arabian center between June 1 and 15, 2020. We measured respiratory mechanics (ventilatory ratio (VR), recruitability index (RI), markers of inflammation, and computed tomography pulmonary angiograms.

**Results:**

Forty-two patients with COVID-19 and 43 non-COVID patients with ARDS comprised the cohort. The incidence of “recruitable” patients using the recruitment/inflation ratio was slightly lower in COVID-19 patients (62 vs. 86%; *p* = 0.01). Fifteen COVID-19 ARDS patients (35.7%) developed a pulmonary embolism as compared to 4 (9.3%) in other ARDS patients (*p* = 0.003). In COVID-19 patients, a D-Dimer ≥ 5.0 mcg/ml had a 73% (95% CI 45–92%) sensitivity and 89% (95% CI 71–98%) specificity for predicting pulmonary embolism. Crude 60-day mortality was higher in COVID-19 patients (35 vs. 15%; *p* = 0.039) but three multivariate analysis showed that independent predictors of 60-day mortality included the ventilatory ratio (OR 3.67, 95% CI 1.61–8.35), PaO2/FIO2 ratio (OR 0.93; 95% CI 0.87–0.99), IL-6 (OR 1.02, 95% CI 1.00–1.03), and D-dimer (OR 7.26, 95% CI 1.11–47.30) but not COVID-19 infection.

**Conclusion:**

COVID-19 patients were slightly less recruitable and had a higher incidence of pulmonary embolism than those with ARDS from other etiologies. A high D-dimer was predictive of pulmonary embolism in COVID-19 patients. COVID-19 infection was not an independent predictor of 60-day mortality in the presence of ARDS.

## Introduction

The worldwide human death toll from the novel severe acute respiratory syndrome coronavirus 2, SARS-CoV-2 disease (COVID-19) has exceeded 302 million. Most people who contract COVID-19 survive, but life-threatening COVID-19 can manifest with acute respiratory distress syndrome (ARDS), multi-system organ failure, venous thromboembolism, and cytokine release syndrome (1–4). COVID-19 patients with ARDS, as defined by the Berlin criteria, currently receive invasive mechanical ventilation and supportive care similar to patients with non-COVID-19 ARDS (5, 6). This study compares respiratory mechanics and laboratory characteristics in patients with ARDS plus COVID-19 vs. those with ARDS but without COVID-19 admitted during the same period.

Initially, two COVID-19-related ARDS phenotypes were suggested: an early L phenotype (low lung elastance, low recruitability), and a late H phenotype (high lung elastance, high recruitability): the latter being reflective of traditional ARDS. Later reports, however, suggested that COVID-19 ARDS patients had similar lung mechanics to patients with ARDS from other etiologies, and similarly, heterogeneous lung recruitability (6–10). It was also proposed that COVID-19 ARDS showed more early disproportionate pulmonary vascular endothelial damage and capillary leak (11). The resultant edema and exudation of proteinaceous fluid into the alveoli causes ventilation-perfusion mismatch, and vascular endothelial damage causes hypoperfusion of oxygenated alveoli (7, 12–15). Conclusions about the respiratory mechanics of COVID-19 ARDS may be complicated by an increased risk of pulmonary embolism (PE), the effects of dexamethasone, and variable use of early proning (16–22). Estimates of dead space, such as the ventilatory ratio (VR), may help in this regard since dead space is a predictor of mortality in ARDS clinical trials but may also be influenced by pulmonary embolism or hyperinflation (23). Potentially, markers like lung recruitability and VR may help clinicians reduce ventilator induced lung injury by maximizing lung unit recruitment and minimizing overdistension (24).

Comparisons of COVID-19 and non-COVID-19 causes of ARDS published so far have used historical controls. The mortality of COVID-19 patients, however, has progressively improved across the time of the pandemic (25). This makes historical comparisons difficult to interpret. We completed a cohort study of patients admitted during the same period in the same center to discern the differences in respiratory mechanics, inflammatory markers, and clinical factors in critically ill patients with ARDS from COVID-19 and other etiologies and understand whether COVID-19 was an independent predictor of mortality in the presence of ARDS. We also investigated predictors of pulmonary embolism.

## Methods

### Selection and Description of Participants

In this cohort study, we enrolled consecutive intubated patients who met criteria for ARDS arising from both COVID-19 and other etiologies. Patients were admitted to the Level-III 300 multi-unit bed Intensive Care Unit (ICU) (King Saud Medical City, Riyadh, Saudi Arabia) between June 1 and June 15, 2020.The King Saud Medical City ICU department is comprised of several subunits including medical, surgical, trauma, burns and neurocritical care and is the largest referral center for trauma in Saudi Arabia. Inclusion criteria were: (1) age ≥ 18 years, (2) requirement for mechanical ventilation, and (3) a diagnosis of ARDS based on the Berlin criteria (5). Exclusion criteria were: (1) intubation for >24 h prior to ICU admission, and (2) transport of a patient to another medical center given a lack of capacity (and not for extracorporeal membrane oxygenation—ECMO). SARS-CoV-2 infection was confirmed or refuted by Real-Time-Polymerase-Chain-Reaction (RT-PCR) assays performed on nasopharyngeal swabs using the Quanti Nova Probe RT-PCR kit (Qiagen, GmbH, Germany) in a Light-Cycler 480 real-time PCR system (Roche, Basel, Switzerland) (26).

The study was conducted according to the principles of the Declaration of Helsinki and approved by our Institutional Review Board.

### Respiratory Mechanics and Lung Computed Tomography Angiography

Mechanical ventilation was delivered to each patient using assist control mode as follows: targets: tidal volumes of 4–6 ml/kg, oxygen saturation (SaO2) of 88–95% and pH of 7.30–7.45. Inspiratory flow rate was 60–80 L/min and patients were prone-positioned for at least 16 h per day. All COVID-19 ARDS patients received prophylactic (non-therapeutic dose) heparinoid anticoagulation, intravenous dexamethasone 6 mg once daily, ribavirin, interferon beta 1b, and empiric antibiotics (21). Similarly all patients with ARDS from other etiologies received prophylactic heparinoid anticoagulation and empiric antibiotics. During the first 48-h after intubation, the ventilator settings, respiratory mechanics, and arterial blood gas values were recorded. Plateau pressures were recorded during a 0.3-second end-inspiratory occlusion, and a 1-to-2 second end-expiratory occlusion was used to determine intrinsic positive end expiratory pressure (PEEP). Putative airway closure was determined by measuring the airway opening pressure (AOP) during a low flow ( ≤ 6 L/min) insufflation (27). The potential for lung recruitment was determined by the mean value of the recruitment-to-inflation (RI) ratio (ratio of the compliance of the recruited lung divided by the compliance of the “baby lung”) using the single-breath drop in PEEP from 15 to 5 cm H2O, as previously described (28). High potential for lung recruitability was indicated by a RI ratio ≥0.5. PaO2/FIO2 ratio was calculated based on standard procedures, as was VR [minute ventilation (ml/min) × PaCO2 (mmHg)]/(predicted body weight x 100 x 37.5) and driving pressure (DP = plateau pressure − PEEP) (23, 29). Heat and moisture exchanger filters were added on all ventilators to minimize any differences in the measured instrumental dead space. Computed tomography pulmonary angiograms (CT-PA) were performed in subjects with a PaO2/FIO2 ratio <80 for > 24 h, and PE were categorized as arising from main/lobar, segmental and sub-segmental lung regions (30).

### Clinical, Laboratory Investigations, and Outcomes

Within the first 24 h of ICU admission we measured the following in both groups: Acute Physiology and Chronic Health Evaluation II (APACHE II), Sequential Organ Dysfunction (SOFA) scores, C-reactive protein (CRP), D-dimers, lactate dehydrogenase (LDH), ferritin, interleukin-6 (IL-6), and neutrophil-to-lymphocyte ratio (31–35). Acute kidney injury was defined using the RIFLE criteria (36). The primary outcome was 60-day mortality and the association of mortality with respiratory mechanics, inflammatory markers, and the etiology of ARDS (COVID-19 vs. other etiologies). Secondary outcomes included the incidence of PE and the association between respiratory compliance, lung recruitability, and PaO2/FIO2 ratio within the two subgroups. Additional outcomes included the association between inflammatory markers and respiratory mechanics.

### Statistical Analysis

Parametric data were presented as mean ± standard error (SE) and non-parametric data were presented as median with interquartile range (IQR) with comparisons being made using the Student's *t*-test or Wilcoxon signed rank test. Categorical variables were expressed as numbers or percentages and compared using Fisher's exact test. Pearson's correlation coefficient (r) measured the association within and between continuous variables. The association between respiratory compliance and either PaO2/FIO2 ratio or RI ratio was performed using linear regression. Pre-specified and significant (*p* < 0.10) variables were fit into three logistic regression models predicting 60-day mortality. Given the small sample size, we limited our logistic models to four variables to minimize bias in the model's parameter estimates as well as the effects of collinearity (37). Kaplan Meier survival functions were used for 60-day survival, stratified by type of ARDS and compared using the log-rank statistic. All tests were two-tailed with a significance *p-*value of <0.05. The analysis was performed using SPSS 25.0 (IBM SPSS Statistics for Windows, Version 25.0, Armonk, NY: IBM Corp.), and STATA 15 (Stata Corp, College Station, Texas).

## Results

### Clinical Characteristics

One hundred and fifteen consecutive patients with ARDS were admitted to the ICU during the observation period. We excluded 10 patients who were intubated for greater than 24 h prior to ICU admission, and 20 who were transported to other ICUs because the existing unit bed capacity was exceeded (not for extracorporeal membrane support—ECMO). No patients had received non-invasive ventilation. The cohort included 42 patients with COVID-19 ARDS and 43 with ARDS from the following etiologies: bacterial pneumonia (*n* = 25), and sepsis syndrome (*n* = 18). There were no significant differences in age, gender, number of comorbidities between patients with COVID-19 and those with ARDS from other etiologies. However, COVID-19 patients had a higher body mass index (BMI), and fewer symptom days prior to ICU admission (Table 1).

**Table 1:** Characteristics of forty-two COVID-19 patients and forty-three patients without COVID-19 and with acute respiratory distress syndrome.

**Parameters**	**All patients (*n =* 85)**	**COVID-19 patients with ARDS (*n =* 42)**	**Non-COVID-19 patients with ARDS (*n =* 43)**	***P*-value**
Age (years)	49.7 ± 0.93	49.5 ± 1.29	49.9 ± 1.36	0.84
Body Mass Index (kg/m^2^)	25.2 ± 0.37	27.1 ± 0.41	23.3 ± 0.46	0.001^*^
Sex (Male, %)	63 (74.1%)	33 (78.6%)	30 (69.8%)	0.22
*Comorbidities, n (%)*				
None One Two or more	43 (50.6%) 24 (28.2%) 18 (21.2%)	22 (52.4%) 12 (28.6%) 8 (19.0%)	21 (48.8%) 12 (27.9%) 10 (23.3%)	0.64 0.92 0.51
Symptoms onset to ICU admission (days)	7.24 ± 0.36	6.1 ± 0.28	8.3 ± 0.62	0.001^*^
SOFA score (baseline)	9.4 ± 0.23	9.7± 0.39	9.2 ± 0.22	0.29
APACHE II score, (baseline)	22.3 ± 0.13	22.4 ± 0.19	22.2 ± 0.72	0.37

*ARDS, acute respiratory distress syndrome; ICU, intensive care unit; APACHE II score, Acute Physiology and Chronic Health Evaluation II score; SOFA score, Sequential Organ Function Assessment score*.

* *P-values ≤ 0.05 were statistically significant (comparisons between the COVID-19 vs. the non-COVID-19 group of patients)*.

### Respiratory Mechanics

**Figure 1:**
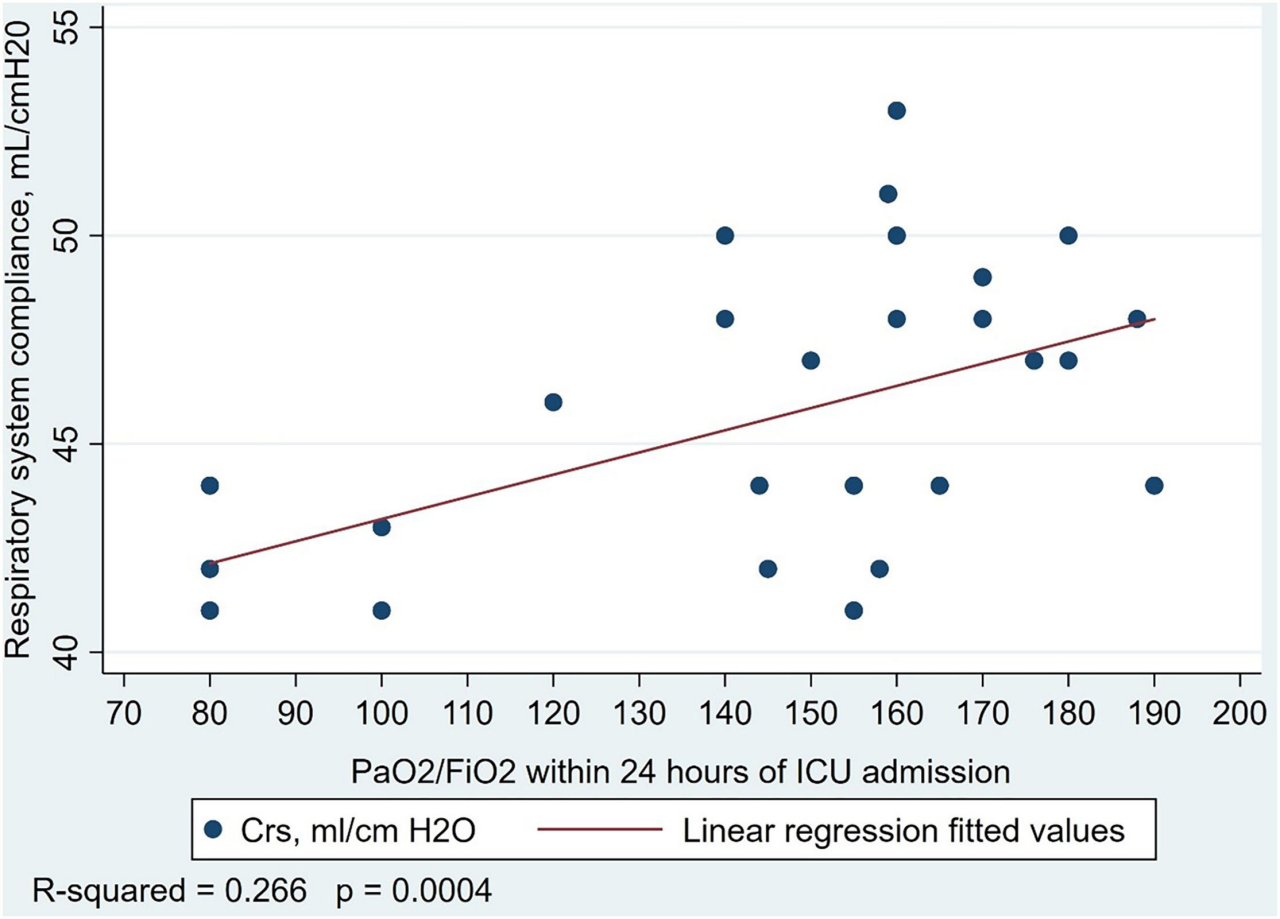
Association between respiratory system compliance and PaO2/FIO2 ratio in 43 patients with acute respiratory distress syndrome from etiologies not related to COVID-19. Linear regression model R2 = 0.266, *P* < 0.001.

With respect to respiratory mechanics, COVID-19 patients were ventilated with higher respiratory rates and lower applied PEEP, and had lower PaO2/FIO2 ratio, and a higher VR than patients without COVID-19. There was no difference in respiratory compliance; the mean plateau pressure and RI ratio was lower in patients with COVID-19 (Table 2). In the COVID-19 patients, fewer patients met the criteria for high recruitability than in other ARDS patients [26 (62%), mean RI ratio 0.58 (0.07), vs. 37 (86%), mean RI ratio 0.59 (0.09); *P* = 0.01]. In patients without COVID-19, there was a linear association between increasing compliance and increasing PaO2/FIO2 ratio (Figure 1). Other associations between respiratory compliance and either PaO2/FIO2 ratio or RI ratio may be found in the Supplementary Figures 1a–c.

**Table 2:** Respiratory mechanics of forty-two COVID-19 patients and forty-three non-COVID-19 patients with acute respiratory distress syndrome.

**Parameters**	**COVID-19 patients with ARDS (*n =* 42)**	**Non-COVID-19 patients with ARDS (*n =* 43)**	***P*-value**
**Ventilatory parameters**			
Tidal volume (ml/kg) of PBW	5.9 ± 0.04	6.1 ± 0.06	0.71
Respiratory rate (cycles/min)	30.8 ± 0.56	28.2 ± 0.62	0.003^*^
Positive-end-expiratory-pressure (cm H_2_O)	10.6 ± 0.25	12.2 ± 0.42	0.002^*^
PaO_2_/FiO_2_ ratio	115.3 ± 5.03	144.7 ± 5.32	0.001^*^
PaO2/FIO2 <100, *n* (%)	16 (38)	26 (41)	0.01^*^
PaO2/FIO2 ≥ 100, *n* (%)	26 (62)	37 (59)	
Respiratory system compliance (ml/cm H_2_O)	45.0 ± 0.50	45.6 ± 0.55	0.46
Respiratory system resistance (cm H_2_O/l/s)	15.5 ± 0.31	15.1 ± 0.44	0.45
Recruitment-to-inflation ratio	0.49 ± 0.02	0.55 ± 0.02	0.04^*^
Ventilatory ratio	1.87 ± 0.05	1.62 ± 0.03	0.001^*^
Plateau pressure (cm H_2_O)	23.8 ± 0.35	25.1 ± 0.33	0.01^*^
Driving pressure (cm H_2_O)	10.1 ± 0.16	10.1 ± 0.21	0.85

*PaO_2_/FiO_2_ ratio, partial arterial pressure of oxygen to fractional inspired concentration of oxygen ratio, Ventilatory ratio = [minute ventilation (ml/min) × PaCO2 (mmHg)]/(predicted body weight ×100 ×37.5), Driving pressure = plateau pressure – PEEP*.

* *P-values ≤ 0.05 were statistically significant (comparisons between the COVID-19 vs. the non-COVID-19 group of patients)*.

### Measures of Inflammation

White blood cell count (WBC) to lymphocyte ratio was over two-times higher, D-dimer over four-times higher, ferritin three-times higher and IL-6 over twenty-times higher in patients with COVID-19 as compared to patients with ARDS from other etiologies (Table 3). The ratio of WBC/Lymphocytes was highly correlated with other inflammatory markers including IL-6 (*r* = 0.84, *P* < 0.0001), D-dimers (*r* = 0.71, *P* < 0.001), ferritin (*r* = 0.58, *P* < 0.001), and CRP (*r* = 0.34, *P* = 0.001). Likewise, values of D-dimers and IL-6 were highly correlated (*r* = 0.72, *P* < 0.0001). Increasing values of markers of inflammation were negatively correlated with PaO2/FIO2 ratio and RI ratio and positively correlated with VR. Correlations between the PaO2/FIO2 ratio and VR with biological parameters are shown in Supplementary Table 1.

**Table 3:** Laboratory parameters of forty-two COVID-19 patients and forty-three non-COVID-19 patients with acute respiratory distress syndrome.

**Laboratory parameters**	**COVID-19 patients (*n* = 42)**	**Non-COVID-19 patients (*n =* 43)**	***P*-value**
Creatinine (mg/dl, normal: 0.6–1.2)	1.05 ± 0.03	1.21 ± 0.05	0.02^*^
White blood cells (cells/mm^3^, normal: 4–10)	13.1 ± 3.5	13.2 ± 2.9	0.84
Lymphocytes (10^9^/l, normal: 1.1–3.2)	0.5 ± 0.2	1.5 ± 0.4	0.001^*^
White blood cells/lymphocytes ratio	29.7 ± 2.44	11.6 ± 1.46	0.001^*^
Platelets (cells/mm^3^, normal: 150–450)	134.5 ± 32.7	156.8 ± 40.8	0.007^*^
International normalization ratio (normal: 0.8–1.2)	1.19 ± 0.30	1.22 ± 0.35	0.70
D-Dimers (mcg/ml, normal: <1)	3.6 ± 0.35	0.76 ± 0.11	0.001^*^
Total bilirubin (μmol/L, normal: 0 to 26)	31.3 ± 1.10	36.4 ± 0.96	0.001^*^
C-reactive protein (mg/L, normal: 0–5)	127.3 ± 15.75	76.4 ± 18.86	0.04^*^
Lactate dehydrogenase (u/L, normal: 100–190)	575.9 ± 57.64	233.4 ± 12.86	0.001^*^
Ferritin (ng/ml, normal: 23–336)	589.1 ± 65.5	190.8 ± 9.94	0.001^*^
Interleukin-6 (pg/ml, normal: 1–7)	353.3 ± 75.56	16.9 ± 6.26	0.001^*^

* *P-values ≤ 0.05 were statistically significant (comparisons between the COVID-19 vs. the non-COVID-19 group of patients)*.

### Complications and Mortality

**Figure 2:**
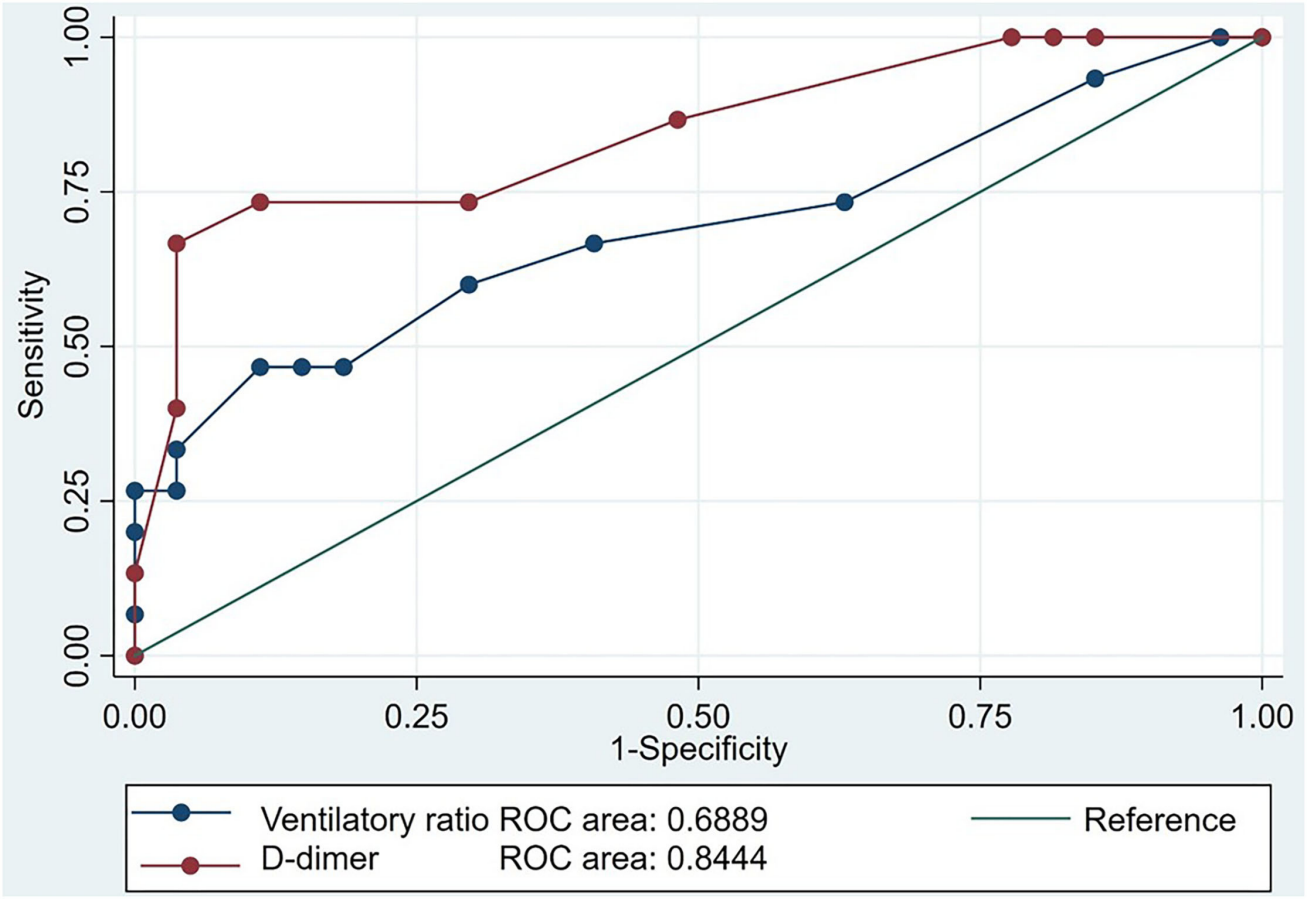
Receiver operator characteristic curve assessing the performance of the ventilatory ratio (VR) and D-dimer in predicting the development of pulmonary embolism in 42 patients with COVID-19 associated acute respiratory distress syndrome. The predictive value of D-dimer was significantly improved over that of the VR when comparing areas under the receiver operator curve (ROC) *P* = 0.03.

As shown in Table 4, COVID-19 patients had a similar prevalence of acute kidney injury but had a higher prevalence of PE than in those with ARDS from other etiologies. Twenty COVID-19 patients and eighteen non-COVID-19 patients underwent CT-PA due to refractory hypoxemia (PaO2/FIO2 ratio <80 for > 24 h). Fifteen COVID-19 patients had a PE, of which 7 were segmental, and 8 subsegmental. This PE prevalence was significantly higher than the 4 patients with ARDS from other etiologies: 3 segmental PEs and 1 sub-segmental; overall 35 vs. 9%, *P* = 0.003. Of patients meeting our criteria for refractory hypoxemia, the prevalence of PE was also significantly higher in the COVID-19 group than in the patients with ARDS from other etiologies; 75 vs. 22.2 %, *P* = 0.003. In patients with COVID-19, the mean VR [2.05 (0.42) vs. 1.77 (0.24), *P* = 0.001] and IL-6 [721.27 (645.44) vs. 148.89 (179.80), *P* < 0.001] were significantly higher in those who developed a PE (Table 4). Inference for PE in patients with ARDS from other etiologies was limited by a low prevalence so the performance of the diagnostic tests in the combined population is outlined in Supplementary Figure 2. However, D-dimer performed significantly better than VR for discriminating the presence or absence of PE in patients with COVID-19 (AUC 0.84, 95% CI 0.71 to 0.98, *P* = 0.03; Figure 2). The sensitivity of a D-dimer ≥ 5.0 mcg/ml in discriminating the presence of absence of a PE in COVID-19 ARDS was 73.3% (95% CI 44.8–92.2%) with a specificity of 88.9% (95% CI 70.8–97.6%). As the performance of a CT-PA (the gold standard for diagnosing a PE) was conditional on meeting the prespecified criteria of a PaO2/FIO2 ratio <80% for > 24 h, we did not evaluate the performance of refractory hypoxemia as a discriminating test nor could we fit this parameter as an independent predictor of PE in our multivariable models. COVID-19 patients had a significantly higher crude 60-day mortality (35 vs. 15%, *P* = 0.039). COVID-19 patients also had shorter durations of mechanical ventilation, ICU length of stay, and hospital length of stay compared to patients with ARDS from other etiologies (Table 5). Shorter lengths of mechanical ventilation and ICU stay were preserved in survivors. Unadjusted Kaplan Meier mean survival of COVID-19 patients (29.64, 95% CI 26.11 to 33.17 days) was significantly shorter than patients with ARDS from other etiologies (48.13, 95% CI 41.31 to 54.94 days, *P* < 001).

**Table 4:** Characteristics associated with the development of pulmonary embolism in COVID-19 patients with acute respiratory distress syndrome.

	**COVID-19**		
**Characteristics**	**Pulmonary embolism present *n =* 15**	**Pulmonary embolism absent, *N =* 27**	***P*-value**
Ventilatory ratio	2.047 (0.4207)	1.767 (0.2434)	*P =* 0.001^*^
RI ratio	0.487 (0.164)	0.496 (0.116)	*P =* 0.83
D-dimer, mcg/mL	5.53 (2.07)	2.65 (1.62)	*P =* 0.23
IL-6, pg/ml	721.27 (645.44)	148.89 (179.80)	*P* < 0.001^*^
CRP, mg/L	145 (122.64)	117.48 (89.767)	*P =* 0.41
Ferritin, ng/ml	661.067 (546.41)	549.22 (345.52)	*P* = 0.42
LDH, u/L	443.4 (348.087)	649.556 (372.877)	*P =* 0.09

* *P-values <0.05 were statistically significant (comparisons between the COVID-19 vs. the non-COVID-19 group of patients)*.

**Table 5:** Complications and outcomes of forty-two COVID-19 patients and forty-three non-COVID-19 patients with acute respiratory distress syndrome.

**Characteristic**	**COVID-19 patients with ARDS (*n =* 42)**	**Non-COVID-19 patients with ARDS (*n =* 43)**	***P*-value**
Mechanical ventilation (days)	19.5 (12.4–24.1)	21.6 (15.9–27.3)	0.004^*^
Survivors (days)	16.8 (12.6–21.0)	21.3 (16.5–25.7)	<0.001^*^
ICU length of stay (days)	21.5 (17.3–28.3)	27.8 (18.2–31.9)	0.001^*^
Survivors (days)	21.2 (16.4–26.0)	26.0 (19.5–32.4)	0.002^*^
Hospital length of stay (days)	30.8 (22.9–37.3)	33.2 (27.8–44.8)	0.001^*^
Acute kidney injury, *n* (%)	4 (9.5)	7 (8.2)	0.35
Pulmonary embolism, *n* (%)	15 (35.7)	4 (9.3)	0003^*^
60-day mortality, *n* (%)	15 (35.7%)	6 (14%)	0.02^*^

*ICU, intensive care unit. Acute Kidney Injury as defined by the RIFLE criteria*.

* *P-values ≤ 0.05 were statistically significant (comparisons between the COVID-19 vs. the non-COVID-19 group of patients)*.

In predicting 60-day mortality, APACHE II score was collinear with VR, and VR was collinear with D-dimer and IL-6. Given the small number of patients and collinearity between these variables we fit three separate models to avoid biased estimates of our model parameters. Independent predictors of mortality using Model 1 were, an increasing VR (OR 3.67, 95% CI 1.61 to 8.35, *P* = 0.002) and a decreasing PaO2/FIO2 ratio (OR 0.93, 95% CI 0.87 to 0.99, *P* = 0.02). Increasing IL-6 (OR 1.02, 95% CI 1.00 to 1.03, *P* = 0.047) and D-dimer (OR 7.26, 95% CI 1.11 to 47.30, *P* = 0.04) also independently predicted mortality in models which included respiratory parameters and ARDS etiology (Model 2 and 3, Supplementary Table 2). Having ARDS from other etiologies independently predicted mortality only at the threshold level of significance (*P* = 0.05) in only one model, and COVID-19 infection was not predictive of mortality in any of the models (Table 6, Supplementary Table 2).

**Table 6:** Univariate and multivariate logistic regression analysis of predictors of 60-day mortality in eighty-five COVID-19 and non-COVID-19 patients with acute respiratory distress syndrome.

**Characteristic**	**Univariate odds ratio (95% CI)^†^**	***p*-value**	**Model 1 odds ratio (95% CI)^†^**	***P*-value**
APACHE II	3.31 (1.87–5.87)	<0.001^*^		
Non-COVID-19 ARDS	0.29 (0.10–0.85)	0.024^*^	1.08 (0.06–19.22)	0.05^*^
Respiratory compliance, ml/cm H20	0.82 (0.69–0.97)	0.021^*^	0.65 (0.41–1.04)	0.07
Ventilatory ratio, per 0.10 units	3.03 (1.79–5.11)	<0.001^*^	3.67 (1.61–8.35)	0.002^*^
PaO_2_/FiO_2_ ratio	0.97 (0.96–0.99)	<0.001^*^	0.93 (0.87–0.99)	0.02^*^
D-Dimer (mcg/ml, normal: <1)	1.84 (1.40–2.43)	<0.001^*^		
Interleukin-6 (pg/ml, normal: 1–7)	1.01 (1.00–1.01)	0.001^*^		

*APACHE II score, Acute Physiology and Chronic Health Evaluation II score; PaO2/FiO2 ratio, partial arterial pressure of oxygen to fractional inspired concentration of oxygen ratio. Ventilatory ratio = [minute ventilation (ml/min) × PaCO2 (mmHg)]/(predicted body weight ×100 ×37.5)*.

* *P-values <0.05 were statistically significant (comparisons between the COVID-19 vs. the non-COVID-19 group of patients)*.

† *CI indicates the 95% confidence interval*.

## Discussion

On average, COVID-19 ARDS patients had comparable respiratory mechanics but differing inflammatory markers compared to patients with ARDS from other etiologies. In COVID-19 ARDS, average recruitability was lower than in other ARDS. However, in patients with COVID-19, higher recruitability was associated with increasing compliance and was independent of the PaO2/FIO2 ratio. These findings may indicate that higher recruitabiliy is achievable in less damaged alveolar lung units with higher baseline compliance. The association between compliance and recruitability was not present in our patients with ARDS from other etiologies possibly because of more heterogeneous pulmonary pathology in this subgroup of ARDS patients. This would suggest that more non-COVID-19 ARDS patients would be required to demonstrate any true association between baseline compliance and recruitability because of increased variability in the underlying pathology. The early phase of ARDS is characterized by alveolar edema and filling by proteinaceous fluid concomitant with the destruction of surfactant producing Type-II alveolar cells. Both alveolar filling with fluid and the reduction in surfactant production results in reduced static respiratory system compliance in injured segments of the lung. Recruitability is largely attributable to an increase in end expiratory lung volume from increases in aerated alveoli with recruitement (38, 39). We found a high proportion of high recruitability in both ARDS subgroups reflective of some preservation of normal alveolar units. However, in our study a significantly lower proportion of COVID-19 ARDS patients had high recruitability. COVID-19 can activate the coagulation cascade, cause vascular endothelial damage, disrupt pulmonary vasoregulation, and create early ventilation-perfusion mismatch and shunt through a mechanism of capillary leak and pulmonary edema resulting in a reduction in static respiratory system compliance. Concomitantly, COVID-19, via its disruption of pulmonary vasoregulation, can also increase dead space in non-perfused alveoli (7, 11, 13, 14, 40). Our findings that 62% of COVID-19 ARDS patients were highly recruitable aligns with previous French and Italian studies that reported 64% and 73% (respectively) of COVID-19 ARDS patients as being highly recruitable (9, 10). Our findings differ from those of an earlier Chinese and another French study where only 17 and 30% of COVID-19 patients were highly recruitable (6, 8). Although highly recruitable lung units should be responsive to an increase in PEEP, our population of COVID-19 ARDS patients had significantly less mean PEEP delivered within the first 48 h than did our patients with ARDS from other etiologies. This may be attributable to the fact that significantly fewer COVID-19 ARDS patients were deemed to be highly recruitable and consequently, an escalation of PEEP was not performed. The fact that the mean VR was higher and PaO2/FIO2 ratio was lower in the COVID-19 ARDS group may also indicate that the higher dead space ventilation in the COVID-19 ARDS group would not have been reduced with increasing PEEP levels.

Ventilatory ratio was significantly higher in COVID-19 ARDS than in ARDS from other etiologies. Ventilatory ratio was also associated with the presence of PE in COVID-19 patients, and it was an independent predictor of mortality in the entire cohort. As VR is only a respiratory physiological parameter indicating the degree of dead space ventilation it may only be viewed as a physiological surrogate of disease severity and not as a biological or pathological process characterizing ARDS in either subgroup. In addition, in the COVID-19 subgroup, D-dimer performed better than the VR in discriminating the presence or absence of PE in those patients with refractory hypoxemia who mandatorily received a CT-PA.

We found higher VR in COVID-19 ARDS. This aligns with the findings of Grieco et al. (9). Our findings highlight the importance of VR as an independent predictor of both PE and mortality in COVID-19 ARDS, and duplicates both a recent Italian cohort of COVID-19 patients, and studies of patients with ARDS from other etiologies (3, 23, 41, 42). We also determined a good sensitivity and specificity for diagnosing PE in COVID-19 ARDS by using a D-dimer threshold level of ≥ 5 mcg/ml. These findings align with those of an Italian cohort which showed that higher D-dimer levels predicted PE and mortality in COVID-19 ARDS (3).

Illness severity was comparable between groups, but COVID-19 patients had a higher crude mortality. In the entire cohort, a higher VR and a lower PaO2/FIO2 ratio as well as higher IL-6 and D-dimer levels were independent predictors of 60-day mortality after adjusting for baseline compliance and etiology (subgroup) of ARDS. This suggest that markers of inflammation are strongly associated with the underlying pathophysiology of both ARDS subgroups. Higher levels of inflammatory markers and incident PE were demonstrated in the COVID-19 subgroup, and inflammatory markers were positively associated with incident PE, predominantly in the COVID-19 ARDS patients. Although our findings do not suggest causality, other authors have suggested that inflammation and vasoconstriction resulting in microthrombosis are important factors in COVID-19 ARDS (43, 44). Microthrombosis and extension of this thrombotic process into sub-segmental and segmental pulmonary arteries may account for the findings of these distal pulmonary arteries being predisposed to thrombosis as compared to proximal pulmonary thrombosis seen in non-COVID-19 diseases. Previous authors have described a predominance of CT-PA confirmed thrombus involving distal pulmonary vessels and located in lung parenchymatous condensations in COVID-19 patients with pulmonary embolus (45).

Given the high prevalence of PE in COVID-19 ARDS, and its associated mortality, our study could encourage early use of VR and D-Dimer as discriminatory tests, confirmation with a CT-PA, and empiric anticoagulation in circumstances where a CT-PA may not be able to be performed safely (43, 46–48). However, our findings of a 75% PE prevalence in those COVID-19 ARDS patients with a PaO2/FIO2 ratio <80 for > 24 h may question the additional usefulness of the VR or a D-Dimer in deciding on empiric therapeutic anticoagulation. We did not perform a CT-PA in less hypoxemic patients where the prevalence of PE may have been lower and the discriminatory value of VR or D-Dimer may have been higher. We also demonstrated elevated laboratory investigations in COVID-19 ARDS: including WBC/lymphocyte ratio, D-Dimer, C-reactive protein, LDH, ferritin, and IL-6. Although the importance of D-Dimer and IL-6 as predictors of COVID-19 mortality has been noted by others, ours is the first study to confirm the robustness of this association independent of respiratory mechanics variables such as VR and PaO2/FIO2 ratio (3, 34, 46, 49).

A strength of our study was the contemporaneous inclusion of all patients and the use of a uniform strategy for mechanical ventilation. Also, all patients with COVID-19 received a standard dose of dexamethasone, which differ from most previous reports. Our study has limitations including its small sample size, and the fact that we did not directly measure dead space, nor work of breathing prior to intubation. Consequently, we could not conclude whether patients would develop self-inflicting lung injury (50). We also did not sequentially measure respiratory mechanics, so we could not characterize temporal improvement or deterioration. Despite limitations, we did demonstrate that a bedside measurement, VR, predicts both PE and mortality. We have also reaffirmed, although not causal, the predictive value of high D-Dimer and IL-6 levels in predicting the development of PE and mortality in COVID-19. This bolsters the rationale behind clinical studies into IL-6 targeted immunomodulatory therapies for severe COVID-19 (4, 51, 52). However, the significant associations we found between inflammatory markers and respiratory physiological parameters and mortality are in no way causal in nature. Such associations are only hypothesis generating and require further pathophysiological investigations into the biological mechanisms linking these markers to vascular and alveolar injury and death.

## Interpretation

In conclusion, in addition to illness severity and PaO2/FIO2 ratio, VR, D-Dimer and IL-6 were independent predictors of mortality in COVID-19 ARDS. D-Dimer at a threshold of ≥ 5 mcg/mL has good sensitivity and specificity in discriminating the presence or absence of PE as confirmed in ARDS patients with refractory hypoxemia. A high proportion of our COVID-19 ARDS patients had high recruitability in whom both oxygenation and ventilation should improve with higher PEEP. In the presence of ARDS we did not find, however, that COVID-19 was an independent predictor of mortality.

## Implication Statement

In a contemporaneous cohort of patients with COVID-19 associated ARDS and ARDS from other etiologies, COVID-19 patients were slightly less recruitable and had a higher level of inflammatory markers and incidence of pulmonary embolism. However adjusted mortality did not differ between groups.

## Data Availability Statement

The raw data supporting the conclusions of this article will be made available by the authors, without undue reservation.

## Ethics Statement

The studies involving human participants were reviewed and approved by Kingdom of Saudi Arabia Ministry of Health, King Saud Medical City. IRB registration number with KACST, KSA: H-01-R-053. IRB registration number U.S. Department of HHS IORG #: IORG0010374. The patients/participants provided their written informed consent to participate in this study.

## Author Contributions

DKu, AA, AB, FF, JP, SA, ZM, and DKa contributed to the conception and design, drafting and revision, interpretation, and final approval of the study and manuscript. PB and LB contributed to the drafting, revision, interpretation, and final approval of the study manuscript. DKu contributed to the statistical analysis of the study. All authors contributed to the article and approved the submitted version.

## Conflict of Interest

The authors declare that the research was conducted in the absence of any commercial or financial relationships that could be construed as a potential conflict of interest.

## Publisher's Note

All claims expressed in this article are solely those of the authors and do not necessarily represent those of their affiliated organizations, or those of the publisher, the editors and the reviewers. Any product that may be evaluated in this article, or claim that may be made by its manufacturer, is not guaranteed or endorsed by the publisher.

## Abbreviations

ARDS: Acute respiratory distress syndrome
AUC: Area under the receiver operator curve
COVID-19, Severe acute respiratory syndrome coronavirus 2: SARS-CoV-2
CRS: Cytokine release syndrome
CT-PA: Lung computed tomography pulmonary angiography
PaO2/FIO2 ratio: Ratio of arterial oxygen partial pressure to fractional inspired oxygen
PE: Pulmonary embolism
RI: Lung recruitability index
RIFLE, Risk, injury: failure criteria for acute kidney injury
ROC: Receiver operator characteristic curve
VR: Ventilatory ratio
WBC: White blood cell count.
