# Supplementary material for: Mortality and Pulmonary Embolism in Acute Respiratory Distress Syndrome From COVID-19 vs. Non-COVID-19
Source: Front Med (Lausanne). 2022 Mar 4;9:800241. doi: 10.3389/fmed.2022.800241 (PMC8931188; doi:10.3389/fmed.2022.800241)
Supplement: Supplementary file 1 [file Data_Sheet_1.docx]

**Mortality and pulmonary embolism in Acute Respiratory Distress Syndrome from COVID-19 versus**

**non-COVID-19**

Demetrios J. Kutsogiannis, MD ^1^; Abdulrahman Alharthy, MD ^2^; Abdullah Balhamar, MD ^2^; Fahad Faqihi, MD ^2^; John Papanikolaou, MD ^2^; Saleh A. Alqahtani, MD ^3^; Ziad A. Memish, MD ^4^; Peter G. Brindley, MD ^1^; Laurent Brochard, MD ^5,6,7^; Dimitrios Karakitsos, MD ^2,8,9^

1. Department of Critical Care Medicine, Faculty of Medicine and Dentistry, the University of Alberta, Edmonton, Alberta, Canada.

2. Critical Care Department, King Saud Medical City, Riyadh, Saudi Arabia.

3. Department of Medicine, The Johns Hopkins University Hospital, Baltimore, MD, USA.

4. Research and Innovation Center, King Saud Medical City, Riyadh, Saudi Arabia.

5. Keenan Research Center and Li Ka Shing Institute, Department of Critical Care, St. Michael’s Hospital, Toronto, Ontario, Canada

6. Interdepartmental Division of Critical Care Medicine, and Institute of Medical Science, University of Toronto, Toronto, Ontario, Canada

7. Institute of Medical Science, University of Toronto, Toronto, Ontario, Canada

8. Department of Internal Medicine, University of South Carolina, School of Medicine, Columbia, SC, USA.

9. Critical Care Department, Keck School of Medicine, University of Southern California, Los Angeles, CA, USA.

Corresponding author: Demetrios J. Kutsogiannis [djk3@ualberta.ca](mailto:djk3@ualberta.ca)

**Supplementary Appendix**

**
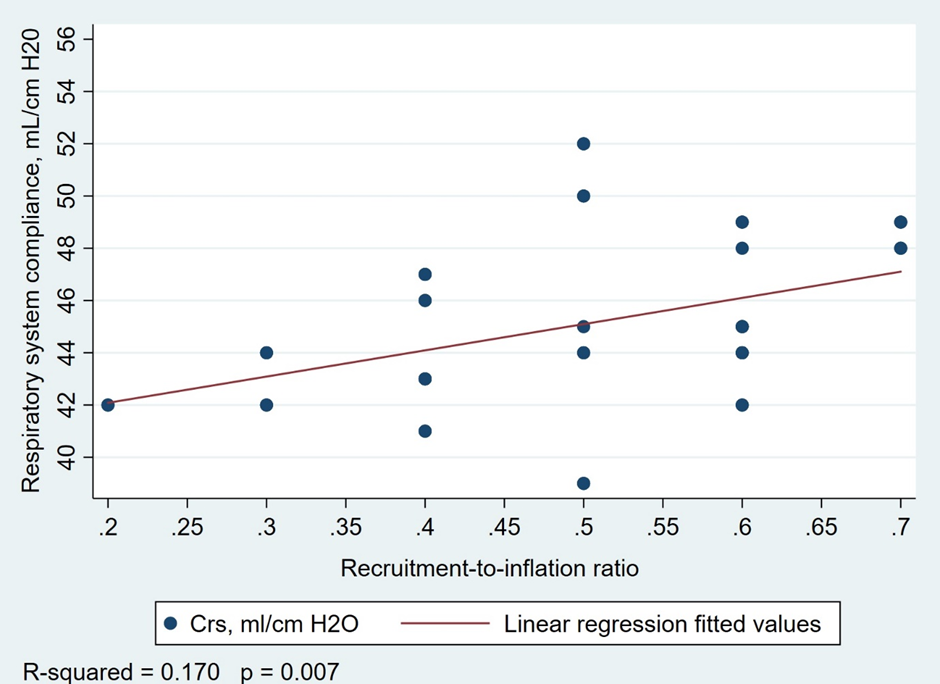
**

**e-Figure 1a.** Association between respiratory system compliance (ml/cm H20) and the recruitment-to-inflation ratio (RI ratio) in 42 patients with COVID-19 and suffering from ARDS.

**
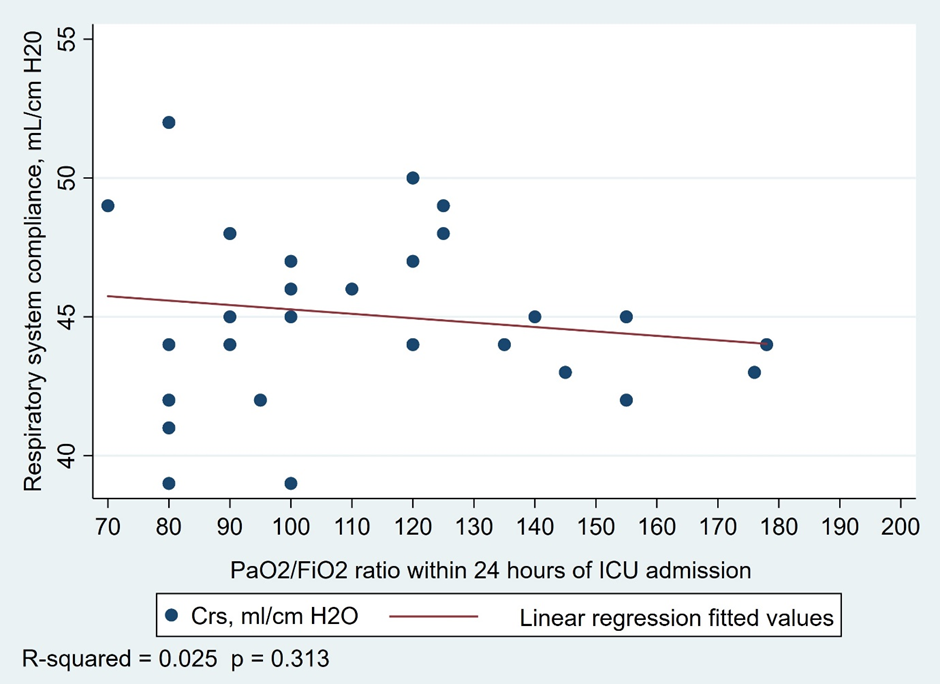
**

**e-Figure 1b.** Association between respiratory system compliance and PaO2/FIO2 ratio in 42 patients with COVID-19 and suffering from ARDS.

**
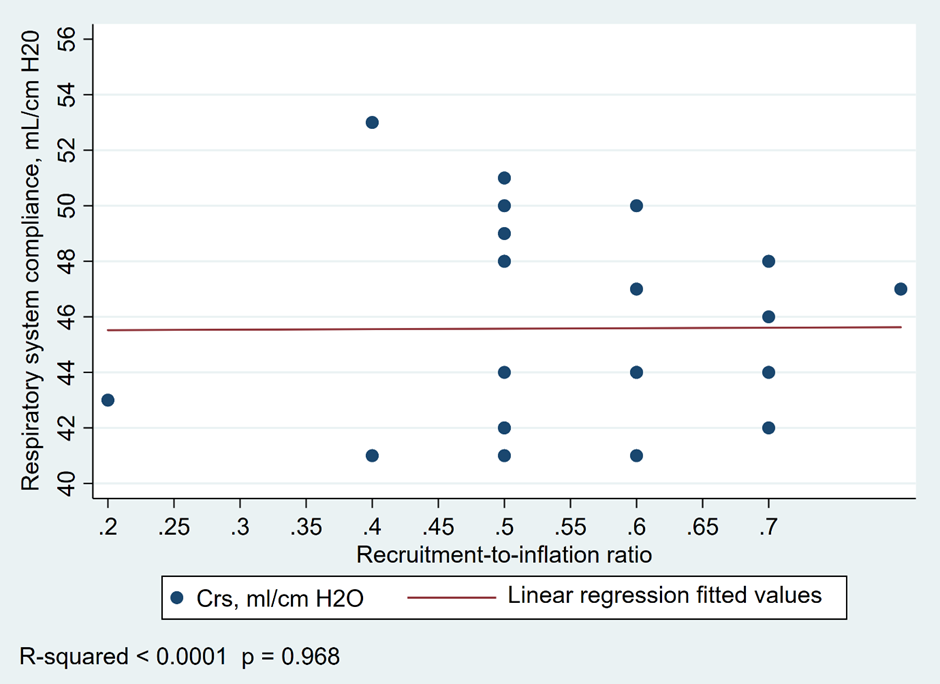
**

**e-Figure 1c.** Association between respiratory system compliance and recruitment-to-inflation ratio in 43 patients with ARDS from etiologies not related to COVID-19.


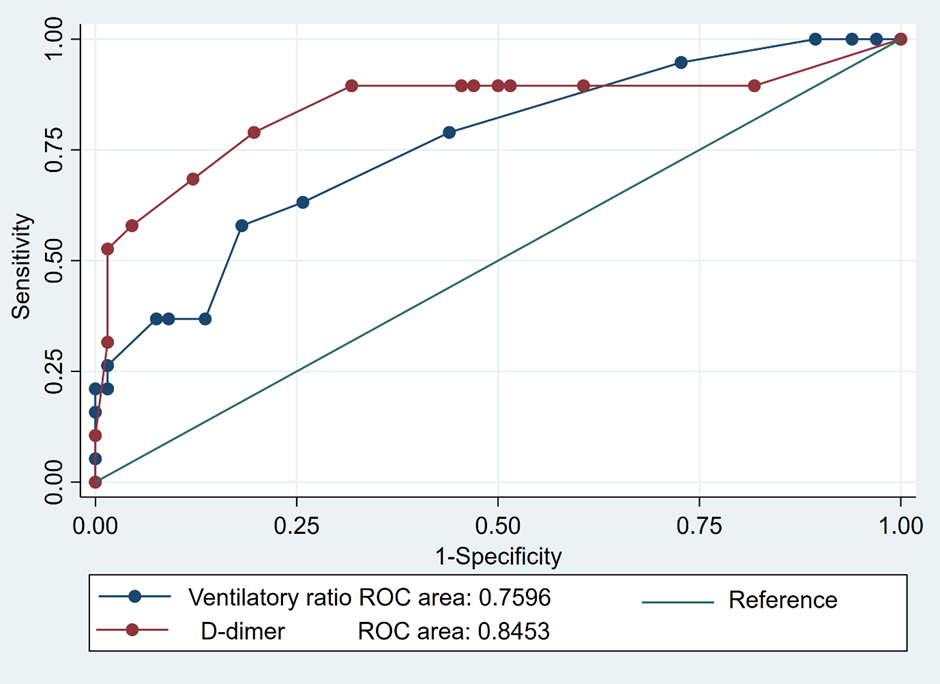


**e-Figure 2.** Receiver operator characteristic curves assessing the performance of the ventilatory ratio (VR) and D-dimer in predicting the development of pulmonary embolism in the combined cohort of 85 patients with COVID-19 and ARDS of other etiologies. The area under the receiver operator curve was larger using D-dimer as a diagnostic test for pulmonary embolism however it did not achieve statistical significance, P = 0.24.

|  | **WBC/Lymphocyte** | **CRP, mg/l** | **Ferritin, ng/ml** | **D-dimers mcg/ml** | **IL-6, pg/ml** | **PaO2/FIO2** | **Compliance, ml/cm H20** |
| --- | --- | --- | --- | --- | --- | --- | --- |
| **WBC/Lymphocyte, cells/mm^3^** | 1.000 |  |  |  |  |  |  |
| **CRP, mg/l** | 0.2712  (0.01)* | 1.000 |  |  |  |  |  |
| **Ferritin, ng/ml** | 0.5785  (<0.001)* | 0.2812  (0.009)* | 1.000 |  |  |  |  |
| **D-dimers, mcg/ml** | 0.7135  (<0.001)* | 0.3753  (<0.001)* | 0.6507  (<0.001)* | 1.000 |  |  |  |
| **IL-6, pg/ml** | 0.8381  (<0.001)* | 0.3064  (0.004)* | 0.6233  (<0.001)* | 0.7174  (<0.001)* | 1.000 |  |  |
| **PaO2/FIO2** | -0.3572  (<0.001)* | -0.2190  (0.04)* | -0.4717  (<0.001)* | -0.4931  (<0.001)* | -0.4208  (<0.001)* | 1.000 |  |
| **Compliance**  **ml/cmH20** | -0.1738  (0.11) | 0.0817  (0.46) | -0.2335  (0.03)* | -0.1989  (0.07) | -0.0570  (0.60) | 0.2262  (0.04)* | 1.000 |
| **RI ratio** | -0.3299  (0.002)* | -0.4151  (<0.001)* | -0.3255  (0.002)* | -0.3932  (<0.001)* | -0.2644  (0.01)* | 0.1610  (0.14) | 0.2122  (0.05)* |
| **VR** | 0.5539  (<0.001)* | 0.2091  (0.05)* | 0.4333  (<0.001)* | 0.5098  (<0.001)* | 0.5237  (<0.001)* | -0.4563  (<0.001)* | -0.1648  (0.13) |
|  |  |  |  |  |  |  |  |
|  |  |  |  |  |  |  |  |
|  | **RI ratio** | **VR** |  |  |  |  |  |
| **RI ratio** | 1.000 |  |  |  |  |  |  |
| **VR** | -0.3809  (<0.001) | 1.000 |  |  |  |  |  |

**e-Table 1.** Correlation between laboratory measures of inflammation and coagulation and respiratory mechanics. Values represent Spearman’s correlation coefficient. Values in brackets represent the P-values of the Spearman’s correlation coefficient.

* P values ≤ 0.05 were statistically significant

| **Characteristics** | **Univariate odds ratio**  **(95% CI) †** | **p-value** | **Model 2**  **Odds ratio**  **(95% CI) †** | **p-value** | **Model 3**  **Odds ratio**  **(95% CI) †** | **p-value** |
| --- | --- | --- | --- | --- | --- | --- |
| **APACHE II** | 3.31 (1.87-5.87) | <0.001 * |  |  |  |  |
| **Non-COVID-19 ARDS** | 0.29 (0.10-0.85) | 0.02 * | 2.28 (0.34-15.11) | 0.39 | 4.67 (0.56-38.95) | 0.16 |
| **Respiratory compliance, ml/cm H20** | 0.82 (0.69-0.97) | 0.02 * | 0.88 (0.69-1.14) | 0.34 | 1.12 (0.81-1.53) | 0.50 |
| **Ventilatory ratio, per 0.10 units** | 3.03 (1.79-5.11) | <0.001 * |  |  |  |  |
| **PaO2/FiO2 ratio** | 0.97 (0.96-0.99) | <0.001 * | (0.95 (0.92-0.99) | 0.007* | 0.95 (0.92-0.99) | 0.007* |
| **D-Dimer, (mcg/ml, normal: < 1)** | 1.84 (1.40-2.43) | <0.001 * |  |  | 7.26 (1.11-47.30) | 0.04* |
| **Interleukin-6, (pg/ml, normal: 1-7)** | 1.01 (1.00-1.01) | 0.001 * | 1.02 (1.00- 1.03) | 0.05* |  |  |

**e-Table 2.** Univariate and multivariate logistic regression analysis of predictors of 60-day mortality in eighty-five COVID-19 and non-COVID-19 ARDS patients.

Definition of abbreviations: APACHE II score: Acute Physiology and Chronic Health Evaluation II score, PaO2/FiO2 ratio: partial arterial pressure of oxygen to fractional inspired concentration of oxygen ratio, Ventilatory ratio = [minute ventilation (ml/min) x PaCO2 (mmHg)] / (predicted body weight x 100 x 37.5).

*P values ≤ 0.05 were statistically significant (comparisons between the COVID-19 versus the non-COVID-19 group of patients).

† CI indicates the 95% confidence interval
